# Anesthesia-related intervention for long-term survival and cancer recurrence following breast cancer surgery: A systematic review of prospective studies

**DOI:** 10.1371/journal.pone.0296158

**Published:** 2023-12-21

**Authors:** Yuecheng Yang, Yunkui Zhang, Yonghong Tang, Jun Zhang

**Affiliations:** 1 Department of anesthesiology, Fudan University Shanghai Cancer Center, Shanghai, China; 2 Department of Oncology, Shanghai Medical College, Fudan University, Shanghai, China; IRCCS: IRCCS Ospedale San Raffaele, ITALY

## Abstract

**Objective:**

Anesthesia is correlated with the prognosis of cancer surgery. However, evidence from prospective studies focusing on breast cancer is currently limited. This systematic review aimed to investigate the effect of anesthesia-related interventions on oncological outcomes following breast cancer surgery in prospective studies.

**Methods:**

Literature searches were performed from inception to June. 2023 in the Pubmed, Web of Science, Embase, and ClinicalTrials databases. The main inclusion criteria comprised a minimum of one-year follow-up duration, with oncological outcomes as endpoints. Anesthesia-related interventions encompassed, but were not limited to, type of anesthesia, anesthetics, and analgesics. The risk of bias was assessed using the Cochrane Risk of Bias Tool.

**Results:**

A total of 9 studies were included. Anesthesia-related interventions included paravertebral nerve block (3), pectoral nerve block (1), sevoflurane (2), ketorolac (2), and infiltration of lidocaine (1). Cancer recurrence, metastasis, disease-free survival, or (and) overall survival were assessed. Among all included studies, only infiltration of lidocaine was found to prolong disease-free survival and overall survival.

**Conclusion:**

Regional anesthesia and propofol did not improve oncological outcomes following breast cancer surgery. The anti-tumorigenic effect of ketorolac warrants future studies with larger sample sizes. Perioperative infiltration of lidocaine around the tumor may be a promising anti-tumorigenic intervention that can prolong overall survival in patients with early breast cancer.

## Introduction

As is well documented, surgical resection remains the gold standard for the treatment of numerous tumors. The influence of anesthesia on cancer recurrence has garnered extensive attention in recent years [1–3]. Preclinical experiments has established that anesthetics and analgesics may potentially impact the prognosis of the cancer [4, 5]. Likewise, a previous meta-analysis reported that propofol can prolong the overall survival of patients with cancer compared with volatile anesthesia [6]. Indeed, compelling evidence suggests that the role of anesthesia in cancer recurrence deserves special attention.

More than 2 million women are diagnosed with breast cancer annually, the five-year mortality rate of which was 4.9% in the last decade (Carolyn *et al*. Corrected in doi: 10.1136/bmj.p1744, original article: doi: 10.1136/bmj-2022-074684) [7]. In recent years, multiple retrospective studies have focused on the role of anesthesia in breast cancer recurrence [8, 9]. A previous systematic review undertaken in 2017 reported that regional anesthesia does not improve oncological outcomes in patients with breast cancer [10]. Nevertheless, studies reporting long-term outcomes in the systematic review were limited. The majority of prospective studies on this topic have been published in the past six years. To the best of our knowledge, there has been no systematic review focusing on the role of anesthesia intervention in breast cancer recurrence and long-term survival.

Therefore, this systematic review of prospective studies was performed to address the gap. Subjects were patients receiving anesthesia-related interventions during breast cancer surgeries whose oncological outcomes were assessed during the follow-up period. The primary objective of this review was to offer valuable insights into the effect of anesthesia-related interventions on breast cancer recurrence.

## Materials and methods

### Search strategy

This systematic review was performed in accordance with the PRISMA guidelines (S1 Text). Two researchers retrieved relevant articles published in English from the Pubmed, Web of Science, Embase, and ClinicalTrials databases from inception to June 2023. Only English language studies were included. The Search terms were as follows: (((trial) AND (((breast cancer) OR (mastectomy)) OR (breast neoplasms))) AND (((long term) OR (survival)) OR (cancer recurrence))) AND ((((anesthesia) OR (anaesthesia)) OR (anesthetic)) OR (analgesic)). The study protocol was registered in INPLASY (No: 202360070, doi: 10.37766/inplasy2023.6.0070). The protocol is detailed in the S1 File.

### Inclusion criteria

The inclusion criteria were as follows: (1) Patients who underwent breast cancer surgeries under anesthesia. (2) Implementation of perioperative anesthesia-related interventions. (3) Data on cancer recurrence and (or) long-term survival as outcomes. (4) A follow-up duration of at least 1 year.

The exclusion criteria were as follows: (1) Animal research studies. (2) Retrospective studies. (3) Case series or reviews.

### Quality assessment

Two independent authors assessed the bias of the included studies using the Cochrane Risk of Bias Tool (Revman 5.4.1). Disagreements were resolved via arbitration with a third author until a consensus was reached. In this assessment, a small sample size (total patients<200) was considered a high risk in “other bias”.

### Data extraction

Data extraction was carried out using two separate forms: characteristics and outcomes. The former consisted of design, intervention, sample size, and follow-up durations. The outcomes form comprised the incidence of cancer recurrence (or metastasis), disease-free survival, and overall survival rate. The data were initially extracted by one author and were subsequently validated by another author.

## Results

### Search results

**Fig 1:**
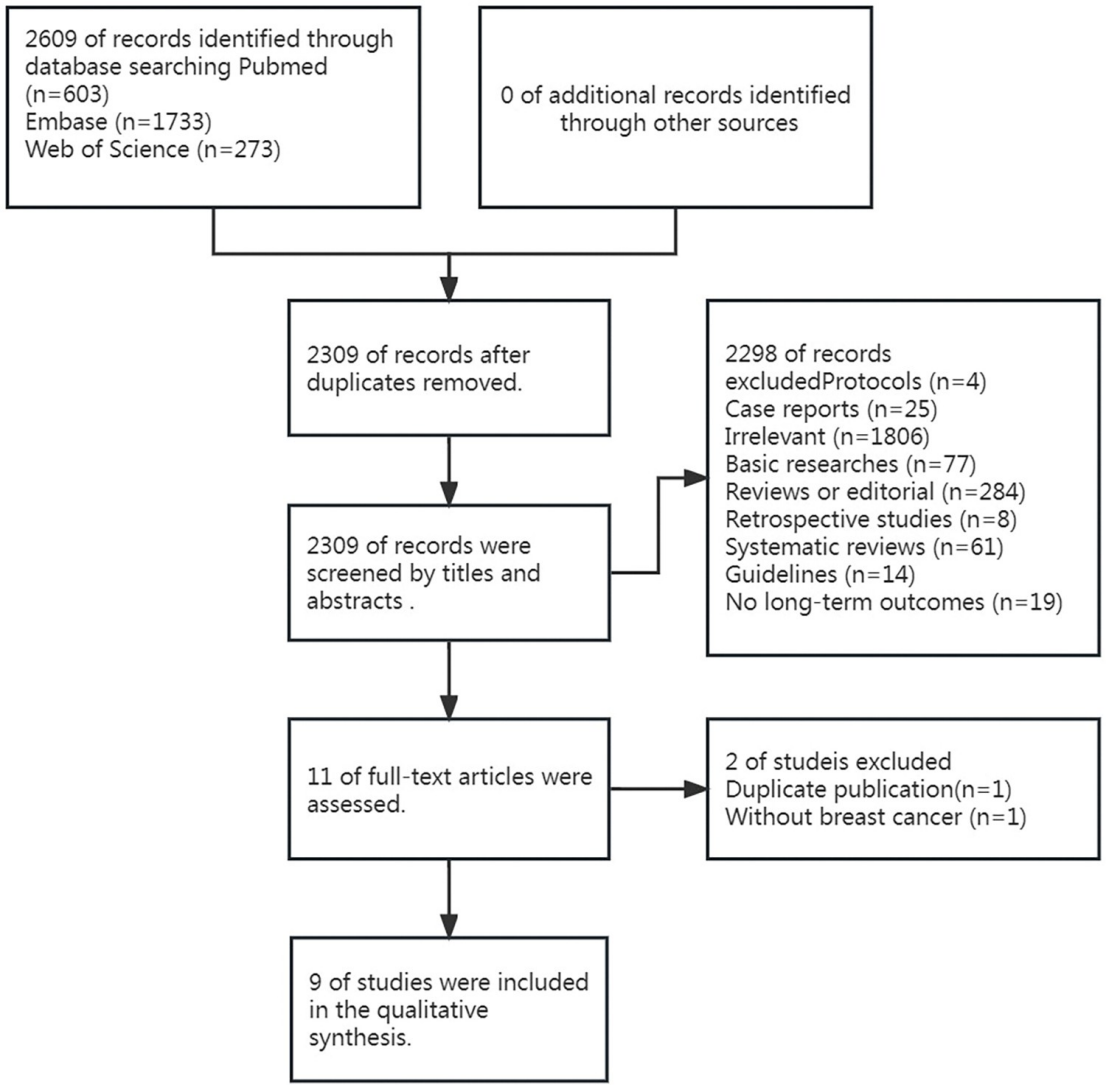
PRISMA flow diagram. Flow chart illustrating the search process.

The pre-defined search yielded 2609 studies, among which 300 duplicates were removed. The titles and abstracts of the remaining 2309 studies were screened, and 2298 studies were excluded for multiple reasons. Next, the full text of 11 studies was meticulously reviewed. One study did not involve breast cancer surgery, whilst another study was identified as a duplicate publication [11, 12]. As a result, 9 studies were finally included (Fig 1). Our search did not extend to Scopus owing to limited access by the authors, which was a deviation from the protocol. Additionally, one trial comparing inhalation and intravenous anesthesia concerning mortality following breast cancer surgery was ongoing, according to the record in ClinicalTrials.gov (NCT04800393). This trial is projected to conclude in 2028.

### Characteristics of studies and patients

All included studies were RCTs and published from 2017 to 2023. Anesthesia-related interventions comprised regional anesthesia (paravertebral nerve block, pectoral nerve block), anesthetics (propofol or sevoflurane), analgesic (ketorolac), and local anesthetic (infiltration of lidocaine). Among the included studies, 3 were multi-center studies (Table 1). Meanwhile, the single-center studies were conducted in the Chinese mainland (2), Hong Kong (1), Korea (1), USA (1), and India (1).

**Table 1:** Design and characteristics of included studies.

Study	Design	Country (Region)	Intervention	Treatment	Control	T Group (n)	C Group (n)	Measured Outcomes	Follow-up duration
Cho 2017 [13]	RCT	Korea	Anesthetics	Propofol + ketorolac	Sevoflurane+fentanyl	24	24	Cancer recurrence	2 years
Finn 2017 [14]	RCT	USA	Type of anesthesia	PB+GA	GA	26	28	Cancer recurrence, disease-free survival, and overall survival	2 years
Karmakar 2017 [15]	RCT	Hong Kong	Type of anesthesia	Single PB+GA and continuous PB +GA	GA	56+59	58	Cancer recurrence and mortality	5 years
Yan 2018 [16]	RCT	China	Anesthetics	Sevoflurane	Propofol +remifentanil	40	40	Cancer recurrence, recurrence-free survival, and overall survival	2 years
Forget 2019 [17]	RCT	Belgium (Multicenter)	Analgesic	Ketorolac	Placebo	96	107	Disease-free survival and overall survival	2 years
Sessler 2019 [18]	RCT	Nine countries	Type of anesthesia + Anesthetics	PB + propofol	Sevoflurane + opioid	1043	1065	Cancer recurrence	Median 36 months
Yu 2022 [19]	RCT	China	Type of anesthesia	Pecs II block +GA	GA	251	252	Disease-free survival and overall survival	5 years
Badwe 2023 [20]	RCT	India	Local anesthetics	0.5% lidocaine infiltration	No placebo	796	804	Cancer recurrence, disease-free survival, and overall survival	Median 68 months
Enlund 2023 [21]	RCT	Sweden, China	Anesthetics	Sevoflurane	Propofol	879	885	Overall survival	5 years

Note: GA: general anesthesia; Pecs II: pectoral nerve block type II

### Study outcomes

Measured outcomes in these studies included cancer recurrence, metastasis, disease-free survival, and overall survival rate. Considering that studies with small sample sizes (total sample size < 200) provided weak evidence, 4 studies were excluded from the outcome analyses (the outcomes of the excluded studies are detailed in S1 Table). In the study conducted by Sessler *et al*, the results of cancer recurrence and metastasis were combined. Oncological outcomes are summarized in Table 2. Among all included studies, only one study reported positive results (infiltration of lidocaine around the tumor) [20].

**Table 2:** Oncological outcomes of included studies.

Study	Cancer recurrence	Metastasis	Disease-free survival rate	Overall survival rate	Conclusion
Forget 2019	/	/	83.1% VS 89.7%	96.8% VS 98.1%	Negative
Sessler 2019	10% VS 10%		/	/	Negative
Yu 2022	/		84.9% VS 81.3%	92.8% VS 91.7%	Negative
Badwe 2023	3.2% VS 4.1%	8.1% VS 10.9%	86.6% VS 82.6%	90.1% VS 86.4%	Positive
Enlund 2023	/		/	92.2% VS 91.9%	Negative

Note: Outcomes were represented as proportion in the treatment group VS proportion in the control group. The conclusions drawn from the studies were reflected in the statistical results. In Sessler 2019, cancer recurrence and metastasis records were combined.

### Risk of bias assessment

**Fig 2:**
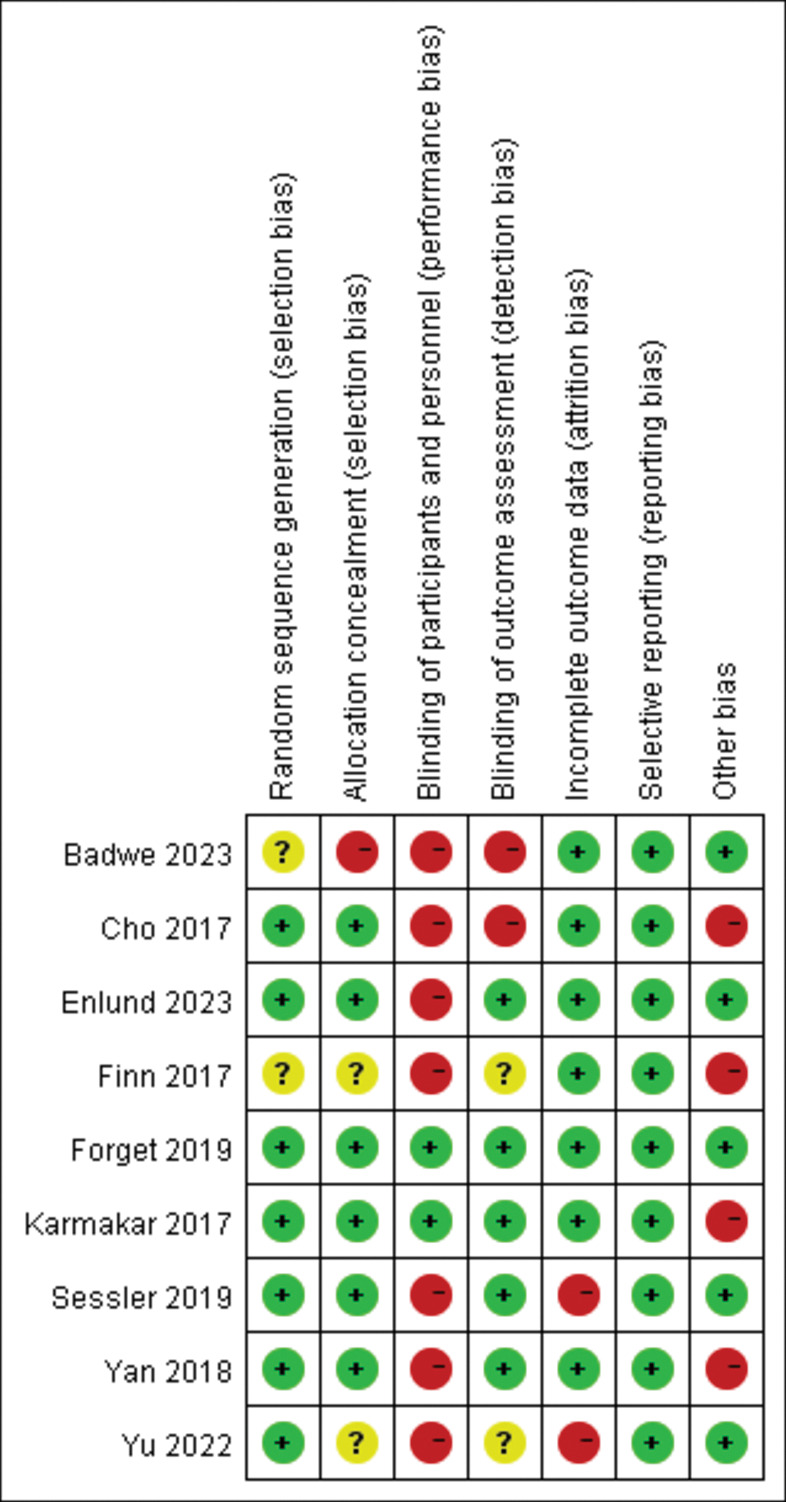
Risk of bias assessment. The risk of bias was assessed using the Cochrane Risk of Bias Tool.

The quality of the included studies is illustrated in Fig 2. One study did not report the method of random sequence generation [20]. Blinding to anesthetists is challenging to implement during the perioperative period. In most studies, investigators who assessed long-term outcomes were blinded. As oncological outcomes were key endpoints in these studies, the risk of selective reporting was low in the included studies. The prognosis after breast cancer surgery requires a large sample size. Therefore, the risk of ‘other bias’ was high in the four studies (sample size < 200).

## Discussion

These included studies employing anesthesia-related interventions, including regional anesthesia, volatile anesthetics, non-steroidal anti-inflammatory drugs (NSAIDs), and local anesthetic. Notably, only local anesthetic infiltration improved clinical outcomes. Meta-analyses were not performed owing to the high heterogeneity in the interventions.

The administration of regional anesthesia may attenuate the immune response during the surgical intervention. The serum of the patients under regional anesthesia was associated with higher human donor natural killer cell cytotoxicity levels [22]. However, previous systematic reviews and meta-analyses failed to establish the clinical significance of regional anesthesia in oncological outcomes [10, 23]. Herein, 3 of 9 prospective studies examined the effect of the paravertebral block on long-term outcomes (2 studies were excluded from the outcome analysis owing to the limited sample sizes). Moreover, a pectoral nerve block was performed in one study. All these studies noted unfavorable results (even in the well-designed, large sample size RCT [18]). Clinical evidence from both retrospective and prospective studies indicated that regional anesthesia may not improve the long-term outcomes of patients who underwent breast cancer surgeries.

Propofol has been documented to possess anti-inflammatory properties [24]. Indeed, a prior meta-analysis of retrospective studies described that propofol significantly improved the overall survival rate compared with volatile anesthesia [6]. Besides, a propensity score-matched study concluded that volatile anesthetics marginally increased the risk of cancer recurrence following colorectal cancer surgery [25]. However, the results were conflicting in studies involving breast cancer surgery [9, 26]. In the current review, two studies focused on the differences between propofol and sevoflurane monotherapies. However, one study was excluded from the analysis due to its small sample size (a total of 80 patients) [16]. The included study evinced that sevoflurane was not associated with worse survival outcomes [21]. Another study (Sessler *et al*.) compared propofol and sevoflurane in conjunction with the use of regional anesthesia. However, long-term cancer recurrence was similar across the groups. Taken together, these results indicated that evidence of the protective role of propofol in breast cancer recurrence is limited. Another ongoing research investigated differences in the mortality rates between propofol and sevoflurane after breast cancer surgery. However, the sample size of that study was merely 130.

Long-term administration of aspirin may mitigate cancer recurrence and improve survival in patients with breast cancer [27, 28]. However, Frisk *et al*. noted that low-dose aspirin did not improve oncological outcomes [29], implying that the dose and frequency of NSAID intake were associated with its anti-tumorigenic effect. Ketorolac is the most frequently used NSAID during the perioperative period. A previous meta-analysis of retrospective studies found that perioperative NSAIDs were associated with superior overall survival after breast cancer surgeries [30]. Furthermore, multiple retrospective studies concluded that intraoperative use of ketorolac reduced the risk of breast cancer recurrence [31, 32]. In the current review, one study investigated the anti-tumorigenic effect of ketorolac [17], and unexpectedly reported negative clinical outcomes. It is important to acknowledge that a major limitation of that study was its relatively small sample size (a total of 203 patients). Buggy *et al*. postulated that the sample size of a similar design study should aim to enroll over 1600 patients if the anticipated survival rate is 30% VS 24 [33]. Given the high survival rate of breast cancer, further studies with larger sample sizes are necessitated in the future.

A large number of studies have corroborated the anti-tumorigenic effect of lidocaine [34–36]. Alexa *et al*. observed that intravenous lidocaine infusion decreases the risk of cancer recurrence in colorectal cancer patients [37]. Notwithstanding, a similar intervention did not improve overall survival rates in patients with pancreatic cancer [38]. Concerning breast cancer, prior investigations have showcased that lidocaine reduced the postoperative expression of neutrophil extracellular trapping [39]. Earlier studies on lidocaine infusion were predominantly designed to investigate pain management in patients with breast cancer rather than oncological outcomes [40, 41]. Herein, only one study administered lidocaine as an intervention to evaluate its anti-tumorigenic effect [20], revealing that peritumoral injection of 0.5% lidocaine significantly prolonged disease-free survival and overall survival rate in patients with early breast cancer; this was the only study reporting positive clinical outcomes in the studies included in this review. This result confirms that intravenous infusion is not the sole method of lidocaine administration. Peritumoral injection of lidocaine was also an easily implemented, low-cost, and promising intervention [42]. However, the study was associated with shortcomings such as: (1) a lack of blinding among surgeons and the researchers assessing follow-up outcomes. (2) Absence of a placebo injection group. These limitations should be addressed in future studies. According to evidence-based medicine, the results of a single RCT are not sufficient to prompt the guideline updates. Therefore, further studies are required to validate the potential anti-tumorigenic effect of lidocaine.

Some limitations of this systematic review cannot be overlooked. To begin, the number of included studies was relatively small. Secondly, the types and stages of breast cancer were not explored, largely due to a lack of subgroup analyses in most studies. Finally, quantitative analysis was be performed due to the high heterogeneity among the studies.

## Conclusion

The existing evidence failed to establish long-term anti-tumorigenic effect exerted by regional anesthesia in breast cancer. Moreover, perioperative administration of NSAIDs did not yield superior oncological outcomes. Lidocaine infiltration appears to be a promising intervention against breast cancer recurrence. Additional high-quality trials (especially concerning local anesthesia) are required in the future.

## Supporting information

S1 Text The PRISMA checklist. (DOC)

S1 File Study protocol of the systematic review. (PDF)

S1 Table Oncological outcomes of the studies with small sample sizes. (DOCX)
