# Supplementary material for: Anesthesia-related intervention for long-term survival and cancer recurrence following breast cancer surgery: A systematic review of prospective studies
Source: PLoS One. 2023 Dec 21;18(12):e0296158. doi: 10.1371/journal.pone.0296158 (PMC10734918; doi:10.1371/journal.pone.0296158)
Supplement: S1 File — (PDF) [file pone.0296158.s002.pdf]

International Platform of Registered Systematic Review and Meta-analysis Protocols

INPLASY

Anesthesia-related Intervention for Long-term Survival and Cancer Recurrence after Breast Cancer Surgery: a Systematic Review of Prospective Trials

INPLASY202360070
doi: 10.37766/inplasy2023.6.0070
Received: 23 June 2023
Published: 23 June 2023

Yang, YC[1]; Zhang, YK[2]; Zhang, J[3].

Corresponding author:
Yuecheng Yang

yang890424@126.com

Author Affiliation:
Fudan University Shanghai Cancer Center.

ADMINISTRATIVE INFORMATION

Support - No funding.

Review Stage at time of this submission - Preliminary searches.

Conflicts of interest - None declared.

INPLASY registration number: INPLASY202360070

Amendments - This protocol was registered with the International Platform of Registered Systematic Review and Meta-Analysis Protocols (INPLASY) on 23 June 2023 and was last updated on 23 June 2023.

INTRODUCTION

Review question / Objective The aim of the review is to investigate the impact of anesthesia-related interventions in breast cancer recurrence and long-term survival in prospective studies.

Condition being studied The influence of anesthesia on cancer recurrence has become a hot topic in recent years. Preclinical experiments showed that anesthetic and analgesic may potentially impact the prognosis of the cancer. However, very few studies reported long-term outcomes in that systematic review. Most prospective studies in this direction were published in the last six years. It is necessary to perform a systematic review of prospective studies on the impact of anesthesia-related intervention on breast cancer recurrence.

METHODS

Participant or population Patients who underwent breast cancer surgeries under general or (and) regional anesthesia.

Intervention Any anesthesia-related interventions during the surgery, including regional anesthesia, specific analgesics or anesthetics.

Comparator Comparator was individualized in different studies. For example, general anesthesia VS regional anesthesia; propofol VS volatile anesthetics; specific analgesic VS placebo.

Study designs to be included Prospective studis (especially RCTs) focusing on the effect of anesthesia on breast cancer recurrence.

Eligibility criteria The inclusion criteria were: (1) Patients received breast cancer surgeries under

Yang et al. INPLASY protocol 202360070. doi:10.37766/inplasy2023.6.0070 Downloaded from https://inplasy.com/inplasy-2023-6-0070/

anesthesia. (2) Perioperative anesthesia-related intervention was used. (3) Cancer recurrence or (and) long-term survival were measured outcomes. (4) The follow-up period was at least 1 year.The Exclusion criteria were: (1) Animal research studies. (2) Retrospective studies. (3) Case series or reviews.

Information sources Pubmed, Web of Science, Embase, Scopus, and ClinicalTrials.

Main outcome(s) Breast cancer recurrence or metastasis in at least one-year follow-up.

Additional outcome(s) Long-term survival.

Quality assessment / Risk of bias analysis Cochrane Risk of Bias Tool was used to assess the bias. Two authors assessed the bias of the included studies independently.

Strategy of data synthesis Outcomes were represented in tables. Meta-analyses were not performed because of high heterogeneity of the interventions.

Subgroup analysis Not applicable.

Sensitivity analysis Not applicable. Meta-analyses were not performed because of high heterogeneity of the interventions.

Language restriction Only English language references were included.

Country(ies) involved China.

Keywords Breast cancer, anesthesia, cancer recurrence.

Contributions of each author

Author 1 - Yuecheng Yang.
Email: yang890424@126.com
Author 2 - Yunkui Zhang.
Author 3 - Jun Zhang.

Yang et al. INPLASY protocol 202360070. doi:10.37766/inplasy2023.6.0070 Downloaded from https://inplasy.com/inplasy-2023-6-0070/
