# Supplementary material for: Anesthesia-related intervention for long-term survival and cancer recurrence following breast cancer surgery: A systematic review of prospective studies
Source: PLoS One. 2023 Dec 21;18(12):e0296158. doi: 10.1371/journal.pone.0296158 (PMC10734918; doi:10.1371/journal.pone.0296158)
Supplement: S1 Table — (DOCX) [file pone.0296158.s003.docx]

S3. Oncological outcomes of the excluded studies

| Study | Cancer recurrence | Metastasis | Disease-free survival | Overall survival | Conclusion |
| --- | --- | --- | --- | --- | --- |
| Cho 2017 | 0% Vs 4.2% | 0% VS 0% | / | / | Negative |
| Finn 2017 | 11.5% VS 7.1% | | 88.5% VS 100% | 84.6% VS 100% | Negative |
| Karmakar 2017 | 1.7 % VS 3.4% | 7.8% VS 8.6% | / | 92.2% VS 94.8% | Negative |
| Yan 2018 | 15% VS 5% | | 78% VS 95% | 97.5% VS 97.5% | Negative |
